# Correlations Between the EGFR Mutation Status and Clinicopathological Features of Clinical Stage I Lung Adenocarcinoma

**DOI:** 10.1097/MD.0000000000001784

**Published:** 2015-10-23

**Authors:** Tetsuya Isaka, Tomoyuki Yokose, Hiroyuki Ito, Masashi Nagata, Hideyuki Furumoto, Teppei Nishii, Kayoko Katayama, Kouzo Yamada, Haruhiko Nakayama, Munetaka Masuda

**Affiliations:** From the Department of Thoracic Surgery (TI, HI, MN, HF, TN, HN); Department of Pathology (TY); Kanagawa Cancer Center Research Institute Cancer Prevention and Control Division (KK); Department of Thoracic Oncology, Kanagawa Cancer Center (KY); and Department of Surgery, Yokohama City University, Yokohama, Kanagawa, Japan (TI, MM).

## Abstract

Advanced lung cancers with epidermal growth factor receptor (EGFR) exon 19 deletions (Ex19s) and EGFR exon 21 L858R point mutations (Ex21s) exhibit different clinical behavior. However, these differences are unclear in resectable primary lung tumors.

The clinicopathological features of 88 (20.9%) Ex19, 124 (29.4%) Ex21, and 198 (46.9%) EGFR wild-type (Wt) clinical stage I primary adenocarcinomas resected between January 1, 2012 and October 31, 2014 were compared by using Chi-square tests, residual error analysis, analysis of variance, and Tukey tests.

Ex21 lesions occurred more frequently in women and never-smokers and had a higher tumor disappearance rate (TDR: 59.6% vs 43.9%; *P* < 0.001) and lower maximum standardized uptake value (maxSUV: 2.0 vs 3.5; *P* < 0.01) than Wt lesions; Ex19 lesions had intermediate values (52.8% and 2.6). There was a low frequency of vascular invasion in Ex21 lesions (12.1%; *P* < 0.05) and a high frequency in Wt lesions (22.7%; *P* < 0.05). Most Ex19 lesions were intermediate-grade adenocarcinoma (lepidic, acinar, and papillary predominant: 73.9%; *P* < 0.05). Wt and Ex21 lesions were predominately high-grade (micropapillary or solid predominant, mucinous variant) and low-grade (adenocarcinoma in situ and minimally invasive adenocarcinoma) adenocarcinoma, respectively. Wt lesions had smaller lepidic components (42.1% vs 56.3%; *P* < 0.001) and larger papillary and solid components (papillary: 15.5% vs 9.0%; *P* < 0.05; solid: 13.2% vs 3.2%; *P* < 0.001) than Ex21 lesions. Most Ex19 lesions had intermediate component rates.

Most Ex21 lesions were low-grade adenocarcinoma with lepidic growth patterns. Wt high-grade adenocarcinomas included solid and papillary components with vascular invasion. Ex19 lesions were intermediate grade between Ex21 and Wt.

## INTRODUCTION

Epidermal growth factor receptor (EGFR) gene mutations occur in 10% to 50% of stage IV nonsmall cell lung cancer. EGFR mutations are frequently observed in adenocarcinomas as well as in Asian women who are nonsmokers.^1–5^ The EGFR status is the most important determining factor for tyrosine kinase inhibitor (TKI) treatment.^2,4,6^ EGFR mutations are found in 30% to 50% of lung adenocarcinomas, with the most common mutations being the EGFR exon 19 deletion (Ex19) and EGFR exon 21 L858R point mutation (Ex21). These 2 mutations account for 85% to 90% of EGFR mutations.^5–8^ TKIs, which bind to the tyrosine kinase domain of EGFR,^7^ have demonstrated clinical efficacy for the treatment of advanced lung adenocarcinoma.^9,10^ Lesions with Ex19 and Ex21 mutations are thought to exhibit different clinical behavior, and some previous reports demonstrated that Ex19 lesions responded better to EGFR-TKIs and platinum-based chemotherapy than Ex21 lesions in a population with unresectable lung cancer.^10–14^

Previous studies have reported differences in clinicopathological features between EGFR mutant (Mt) and EGFR wild-type (Wt) resectable lung adenocarcinomas, including differences in histopathological features,^15–17^ patient prognosis,^15,18,19^ and radiological imaging findings.^20,21^ Most previous studies have indicated that Mt tumors are associated with better prognosis than Wt tumors.^18,19^ Mt tumors have been pathologically characterized in past reports as having a nonmucinous lepidic growth pattern.^15,16,21–24^ However, other studies reported that Mt tumors are highly correlated with a papillary growth pattern,^15,17^ an acinar predominant (AP) pattern,^25^ or a micropapillary growth pattern.^23,26–29^ As the differences between Ex19, Ex21, and Wt tumors remain unclear, we examined the correlations between the EGFR mutation status and clinicopathological features in resected clinical stage I lung adenocarcinoma based on radiological images and histological subtypes according to the International Association for the Study of Lung Cancer/American Thoracic Society/European Respiratory Society International Multidisciplinary Classification of Lung Adenocarcinoma (2011 IASLC/ATS/ERS classification).

## METHODS

### Patients

There were 429 patients who were clinical stage I disease without receiving preoperative treatment or without the evidence of the synchronous multiple lung cancer, between January 1, 2012 and October 31, 2014. Of the 429 patients, the EGFR mutation status was analyzed in 422 patients (98.4%). Of the 422 patients, total 410 patients were enrolled in this study: 88 patients (20.9%) with Ex19 lesions, 124 patients (29.4%) with Ex21 lesions, and 198 patients (46.9%) with Wt lesions. We performed statistical comparisons among these groups. The 12 patients with the other mutation statuses were excluded in this study: 8 patients (1.9%) with exon 18 mutations (G719X), 3 patients (0.7%) with exon 21 mutations (L861Q), and 1 patient (0.2%) with a double mutation.

### Radiological Examinations

Chest computed tomography (CT) images were obtained using an X-Vigor/Real CT scanner or an Aquilion CT scanner (Toshiba Medical Systems, Tochigi, Japan). CT tumor size was determined from high-resolution CT scans with a 1-mm section thickness. The maximum axial tumor size in a pulmonary window level setting (TSPW: level, −600 HU; width, 1600 HU) and tumor size in a mediastinal window level setting (TSMW: level, 40 HU; width, 400 HU) were measured. The tumor disappearance rate (TDR) was calculated based on the following formula: TDR = 1 − (TSMW/TSPW) × 100 (%). Preoperative 18F-fluorodeoxyglucose positron emission tomography/CT scans were obtained in 384 patients (93.7%), and we calculated tumor maximum standardized uptake value (maxSUV). Clinical staging was based on preoperative radiological imaging according to the Union for International Cancer Control, 7th edition, in a joint conference held just before the operation in which thoracic surgeons, pulmonologists, and radiologists participated.

### Pathological Examinations

More than 1 pathologist, including TY, examined all resected specimens and measured pathological tumor size at the place where the tumor appeared to be maximal in size. Sections were stained with Elastica-van Gieson staining to evaluate vascular invasion and pleural invasion, as well as hematoxylin and eosin staining. Lung adenocarcinoma was classified based on the predominant subtype as defined by the 2011 IASLC/ATS/ERS classification and subtyped semiquantitatively by assessing each component in 5% increments.^30^ Based on the predominance of the components, lung adenocarcinoma specimens were categorized as adenocarcinoma in situ (AIS), minimally invasive adenocarcinoma (MIA), invasive adenocarcinoma (InvAd)-lepidic predominant (LP), InvAd-AP, InvAd-papillary predominant (PP), InvAd-micropapillary predominant (MP), InvAd-solid predominant (SP), and InvAd-mucinous variant (MV). Each pulmonary adenocarcinoma subtype was further categorized as low-grade adenocarcinoma (AIS, MIA), intermediate-grade adenocarcinoma (InvAd-LP, InvAd-AP, InvAd-PP), or high-grade adenocarcinoma (InvAd-SP, InvAd-MP, InvAd-MV) based on prognosis, as previously reported by Yoshizawa et al.^31^

### EGFR Mutation Analysis and Statistical Analysis

DNA extraction was performed using the QIAamp DNA FFPE kit (Qiagen K.K., Tokyo, Japan) in accordance with the manufacturer's instructions. Eight sections (thickness, 5–10 μm) were made from a formalin-fixed paraffin-embedded block of tumor tissue, and the section with the largest amount of tumor cells was identified by hematoxylin and eosin staining. The sections were deparaffinized with 1 mL xylene, and the xylene was then removed with 1 mL ethanol (96%–100%). The remaining pellet was resuspended with 180 μL ATL buffer, and incubated with 50 μL proteinase at 56 °C for 1 hours or until the sample was completely lysed. After sequential incubation of the sample at 90 °C for 1 hours, 200 μL AL buffer and ethanol (96%–100%) were added. The lysate was transferred to a QIAamp MinElute Column and centrifuged, and the sample was washed using 500 μL AW1 and 500 μL AW2 buffer. Subsequently, 20 to 100 μL ATE buffer was added to the center of the membrane, followed by incubation at room temperature for 1 minutes. Finally, the sample was centrifuged, and the DNA was collected in new sterile 1.5 mL microcentrifuge tubes.

A fragment method was used to detect Ex19/insertion. Mt genes were amplified by polymerase chain reaction on a Thermal Cycler Dice TP600 (Takara Bio Inc., Shiga, Japan), following which Ex19/insertion was detected by an ABI3130xl Genetic Analyzer (Life Technologies Japan, Tokyo, Japan). To detect exon 18 mutations (G719X), exon 20 mutations (T790 M), and exon 21 mutations (L858R and L861Q), the Cycleave method was used based on the basic principle of real-time polymerase chain reaction. Mt genes were amplified using a Thermal Cycler Dice Real-Time System, TP800 (Takara Bio Inc., Shiga, Japan); the specific sequence of the amplified gene fragment was detected with a high sensitivity after hybridization of chimeric probes for each Mt with the complementary specific sequence. All the patients provided informed consent for all the parts of this study, before the operation. This study was approved by the Institutional Review Board at Kanagawa Cancer Center, and followed the tenets of the Declaration of Helsinki.

We compared continuous variables among the 3 groups using analysis of variance and post hoc comparisons test (Tukey test). Categorical variables were compared among the 3 groups using Chi-square tests. To determine which groups were significantly different after Chi-square tests, we consecutively performed a residual error analysis. All *P*-values less than 0.05 were considered statistically significant.

## RESULTS

Clinical background information for patients with lesions with the 3 EGFR statuses is compared in Table 1. Ex21 tumors were common in women and nonsmokers (*P* < 0.01 and <0.001, respectively), while Wt tumors were common in men and smokers (*P* < 0.001 for both). The TDR was lower in Wt than in Ex21 tumors (43.9% vs 59.6%; *P* < 0.001), and the maxSUV was higher in Wt than in Ex21 tumors (3.5 vs 2.0; *P* < 0.01). There were no significant differences in age, location of the lesion (right or left side), or CT tumor size among the 3 groups. Ex19 tumors had values that were intermediate between Ex21 and Wt with regard to the frequency of male patients, frequency of smokers, TDR, and maxSUV.

**TABLE 1:**
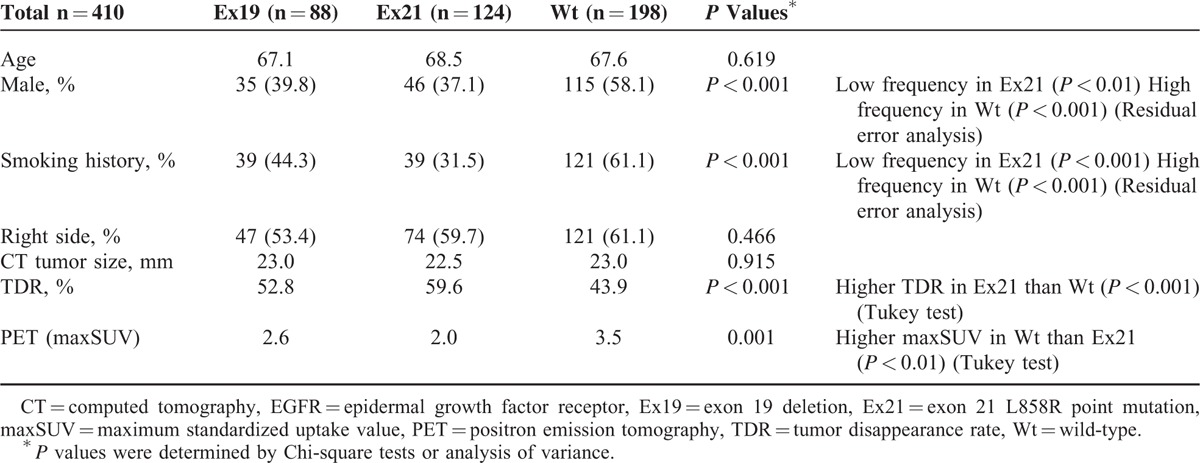
Comparison of Clinical Backgrounds Among Patients With Tumors With the 3 EGFR Statuses

Pathological background information is compared in Table 2. There were no significant differences in pathological tumor diameter, pathological stage, lymphatic invasion, or pleural invasion among the 3 groups. There was a low frequency of vascular invasion in Ex21 lesions (12.1%; *P* < 0.05) and a high frequency in Wt lesions (22.7%; *P* < 0.05). There was a higher frequency of lymph node metastasis in Ex19 lesions (13.6%) than in Wt and Ex21 lesions (9.1% and 5.6%, respectively), although this difference was not significant (*P* = 0.119).

**TABLE 2:**
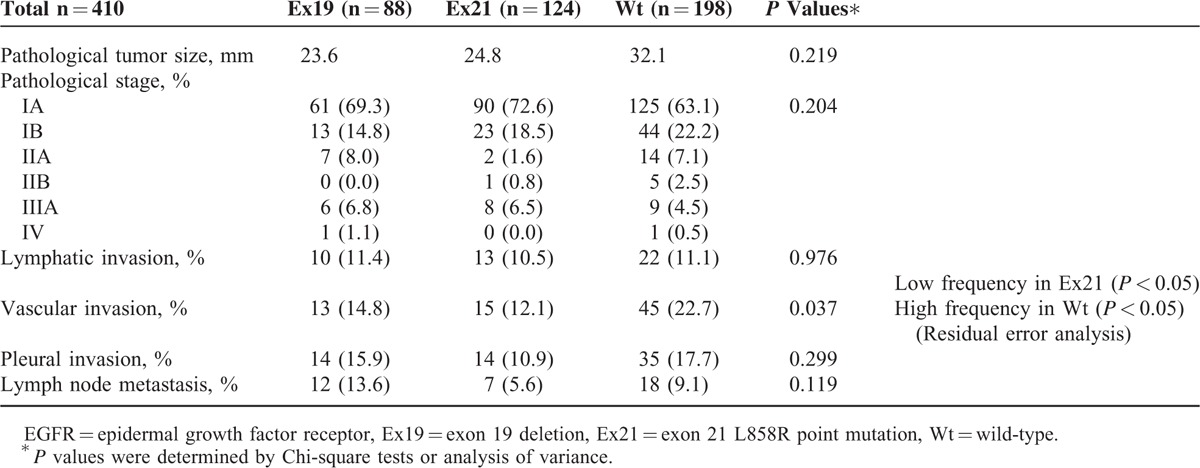
Comparison of Pathological Backgrounds Among Patients With Tumors With the 3 EGFR Statuses

**FIGURE 1:**
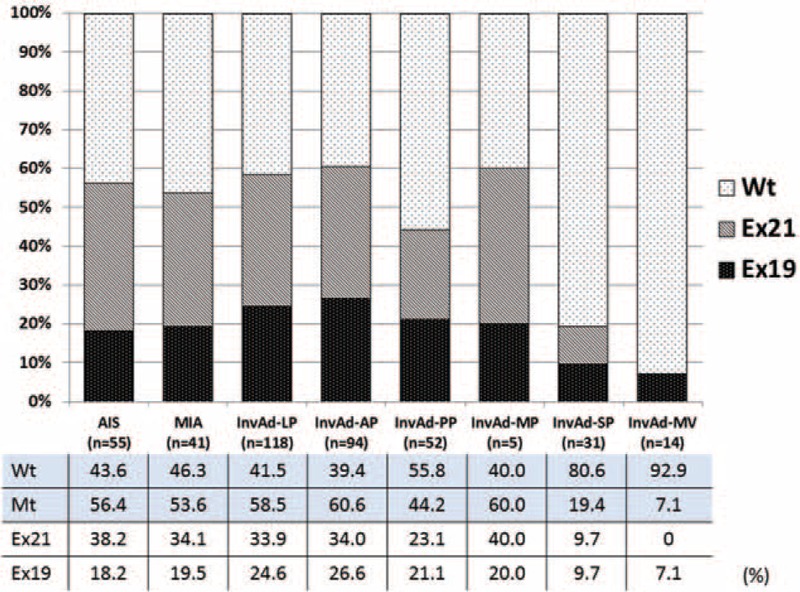
Percentages for the 3 EGFR statuses in each adenocarcinoma subtype based on the 2011 IASLC/ATS/ERS classification. There was a higher percentage of EGFR mutants versus wild-type tumors amongst the adenocarcinoma in situ, minimally invasive adenocarcinoma, invasive adenocarcinoma-lepidic predominant, and invasive adenocarcinoma-acinar predominant specimens. There were approximately twice as many tumors with exon 21 point mutations versus exon 19 deletions amongst the adenocarcinoma in situ and minimally invasive adenocarcinoma specimens. 2011 IASLC/ATS/ERS classification = International Association for the Study of Lung Cancer/American Thoracic Society/European Respiratory Society International Multidisciplinary Classification of Lung Adenocarcinoma, EGFR = epidermal growth factor receptor.

Figure 1 shows the percentage of tumors with each of the 3 EGFR statuses for each adenocarcinoma subtype based on the 2011 IASLC/ATS/ERS classification. Mt tumors more commonly comprised the AIS, MIA, InvAd-LP, InvAd-AP, and InvAd-MP subtypes than the Wt tumors; Ex21 mutations occurred at approximately twice the incidence rate of Ex19 mutations in AIS and MIA subtype tumors.

**FIGURE 2:**
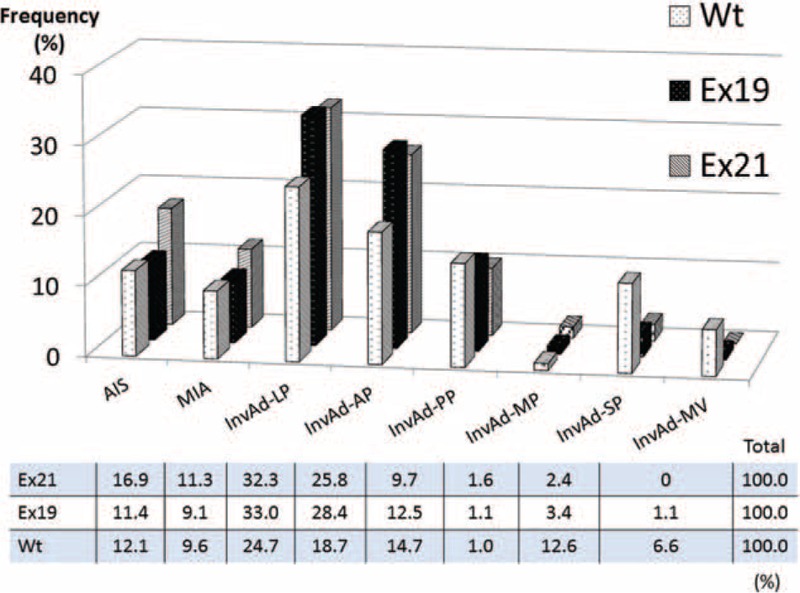
Frequency of each adenocarcinoma subtype based on EGFR status. The frequency of adenocarcinoma in situ and minimally invasive adenocarcinoma was higher in tumors with EGFR exon 21 point mutations (Ex21) compared to tumors with exon 19 deletions (Ex19s) and wild-type (Wt) tumors. The frequency of invasive adenocarcinoma of lepidic and acinar predominant types was higher in both Ex21 and Ex19 tumors than Wt tumors. The frequency of invasive adenocarcinoma that was papillary predominant, solid predominant, and mucinous variant was higher in Wt than EGFR mutant tumors. EGFR = epidermal growth factor receptor, Ex19 = exon 19 deletion, Ex21 = exon 21 L858R point mutation, Wt = wild-type.

Figure 2 shows the frequency of each adenocarcinoma subtype in patients with the 3 EGFR statuses. The frequency of the AIS and MIA subtypes was higher in the Ex21 mutation group (16.9% and 11.3%, respectively) than in the other EGFR status groups. The frequency of InvAd-LP and InvAd-AP was higher in both the Ex21 mutation group (32.3% and 25.8%, respectively) and Ex19 mutation group (33.0% and 28.4%, respectively) than in the Wt group (24.7% and 18.7%, respectively). The frequency of InvAd-PP, InvAd-SP, and InvAd-MV was higher in the Wt group (14.7%, 12.6%, and 6.6%, respectively) than in the Ex19 and Ex21 mutation groups.

**FIGURE 3:**
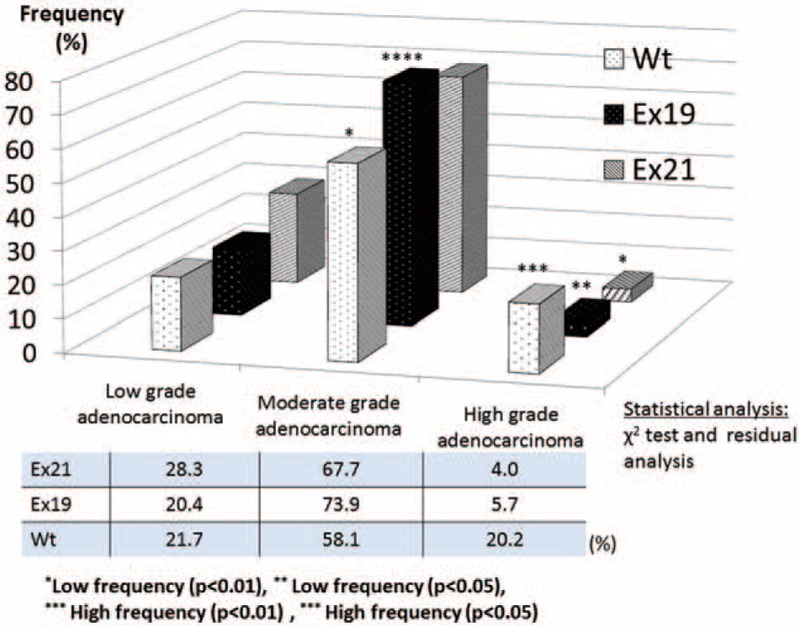
Correlation between pulmonary adenocarcinoma malignancy grade (low/moderate/high-grade) and EGFR status. Pulmonary adenocarcinoma was subdivided into low-grade adenocarcinoma (adenocarcinoma in situ, minimally invasive adenocarcinoma), intermediate-grade adenocarcinoma (invasive adenocarcinoma of lepidic, acinar, and papillary predominant types), and high-grade adenocarcinoma (invasive adenocarcinoma of solid predominant, micropapillary predominant, and mucinous variant types) based on prognosis. There was a high frequency of intermediate-grade adenocarcinoma in the EGFR Ex19 group and a low frequency in the Wt group. There was a high frequency of high-grade adenocarcinoma in the Wt group and a low frequency in the EGFR exon 21 point mutation (Ex21) and Ex19 groups. There tended to be more low-grade adenocarcinoma in the Ex21 group compared to the Ex19 and Wt groups (not significant). EGFR = epidermal growth factor receptor, Ex19 = exon 19 deletion, Ex21 = exon 21 L858R point mutation, Wt = wild-type.

The frequency of intermediate-grade adenocarcinoma was significantly high in the Ex19 mutation group (73.9%; *P* < 0.05) and low in the Wt group (58.1%; *P* < 0.01) (Figure 3). The frequency of high-grade adenocarcinoma was significantly high in the Wt group (20.2%; *P* < 0.01) and low in the Ex21 (4.0%; *P* < 0.01) and Ex19 groups (5.7%; *P* < 0.05). Low-grade adenocarcinoma tended to occur at a higher frequency in the Ex21 group (28.3%) compared to the Ex19 (20.4%) and Wt groups (21.7%).

**FIGURE 4:**
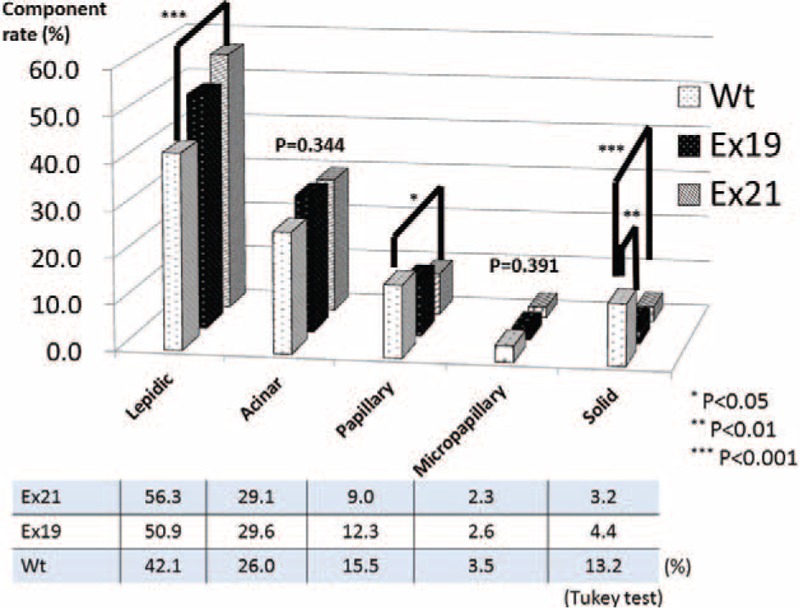
Ratio of each histological component in lung adenocarcinoma specimens. The lepidic component rate was significantly higher in tumors with EGFR exon 21 point mutations (Ex21) than in Wt tumors, and the papillary component rate was higher in Wt than in Ex21 tumors. The solid component rate was higher in Wt tumors than in Ex21 and EGFR Ex19 tumors. The acinar component rate was the highest in Ex19 tumors compared to the other EGFR subtypes; the other adenocarcinoma component rates for Ex19 tumors were intermediate values between the values for Ex21 and Wt tumors. EGFR = epidermal growth factor receptor, Ex19 = exon 19 deletion, Ex21 = exon 21 L858R point mutation, Wt = wild-type.

The lepidic component rate was significantly higher in Ex21 tumors (56.3%) than in Wt tumors (42.1%; *P* < 0.001), while the papillary component rate was higher in Wt tumors (15.5%) than in Ex21 tumors (9.0; *P* < 0.05) (Figure 4). The solid component rate was higher in Wt tumors (13.2%) than in Ex21 (3.2%; *P* < 0.001) and Ex19 tumors (4.4%; *P* < 0.01). The acinar component rate was the highest in the Ex19 group (29.6%; *P* = 0.344). The other adenocarcinoma component rates in the Ex19 group were intermediate values that fell between the rates for the Ex21 and Wt groups.

## DISCUSSION

EGFR is a transmembrane receptor that has intrinsic tyrosine kinase activity and has several specific ligands, such as the epidermal growth factor that binds to EGFR and initiates multiple signaling pathways essential for different cell functions.^2,5^ Mutations of the EGFR genes may result in persistent activation of the tyrosine kinase related to EGFR in tumor cells, and may thus promote tumor proliferation, cell survival, and other cancer-related properties.^5,10,11^ TKI binds to the tyrosine kinase domain to inhibit the protein kinase activity of EGFR, and the EGFR mutation may be the most important predictor of responsiveness to TKI.^4,10,11,13,14^ In recent years, determination of EGFR gene status has played an extremely important role in TKI selection for advanced lung cancer treatment. Exploring the clinicopathological characteristics of lung cancer with EGFR mutations is considered very important for lung cancer treatment. Many studies have reported clinicopathological differences between Wt and Mt tumors, but few studies have examined differences between 2 common EGFR mutations: Ex21 and Ex19. EGFR mutations are frequently found in Asian women who are nonsmokers; they are commonly observed in adenocarcinoma.^1–5^ In this study, Ex21 tumors were more common in women and nonsmokers, compared to Wt tumors, while the frequencies of women and nonsmokers with Ex19 tumors were intermediate between the values for Ex21 and Wt tumors. Ex21 mutations contributed the most to the fact that EGFR mutations were common in women who were nonsmokers.

High maxSUV is associated with high-grade malignancy and poor prognosis of lung cancer patients.^32,33^ Usuda et al^21^ reported that Mt tumors were often small-sized pure or mixed ground-glass opacity lesions with maxSUV lower than Wt tumors. In our study, the maxSUV was the lowest in Ex21 tumors, followed by Ex19 and Wt tumors (Table 1). A low TDR reflected high-grade malignancy and poor prognosis of patients with lung adenocarcinoma. TDR ≥50% indicates noninvasive lung adenocarcinoma with a good prognosis, while TDR <50% indicates invasive lung adenocarcinoma with a poor prognosis and pleural/lymphovascular invasion and lymph node metastasis.^34,35^ In this study, the TDR was the highest in Ex21 tumors, followed by Ex19 and Wt tumors (Table 1). According to the TDR and maxSUV results, Ex21 tumors were more likely to be low-grade malignancies than Wt tumors, while Ex19 tumors were intermediate-grade malignancies that fell between Ex21 and Wt tumors.

Subclassification of resected lung adenocarcinoma based on the 2011 IASLC/ATS/ERS classification correlated with patient prognosis. Yoshizawa et al^31^ described the following categories for tumor grade: AIS and MIA are considered low-grade; InvAd-LP, InvAd-PP, and InvAd-AP are considered intermediate-grade; and InvAd-SP, InvAd-MP, InvAd-MV, and InvAd-colloid predominant are considered high-grade. These classifications are based on the 5-year disease-free survival rates of 100%, 84%, and 71% for low-, intermediate-, and high-grade adenocarcinoma, respectively (*P* < 0.001). Woo et al reported that the 5-year disease-free survival rates for each subcategory of pathological stage I lung adenocarcinoma were 100% for AIS, 100% for MIA, 93.5% for InvAd-LP, 83.7% for InvAd-AP, 75.0% for InvAd-PP, 44.4% for InvAd-SP, and 62.5% for InvAd-MV. High-grade adenocarcinoma including InvAd-SP, InvAd-MP, and InvAd-MV was an independent prognostic factor (hazard ratio, 3.66; *P* = 0.007).^36^ In this study, the Ex21 group included a higher proportion of low-grade adenocarcinoma, whereas the Ex19 group included a higher proportion of intermediate-grade adenocarcinoma, such as InvAd-LP or InvAd-AP. High-grade adenocarcinoma lesions were more frequently Wt tumors instead of Ex19 or Ex21 tumors (Figures 2, 3).

Several studies reported that Mt tumors were mainly composed of the lepidic component,^15,16,21–24^ and it was thought that this was mainly true in Ex21, not Ex19, mutant tumors (Figure 4). The solid component rate was significantly higher in Wt tumors than in Ex19 and Ex21 tumors; this is consistent with previous studies in which adenocarcinoma with a solid histologic subtype was strongly associated with KRAS mutations^37,38^ and was rare in Mt tumors.^15^ Unlike previous studies in which the papillary histologic subtype was associated with Mt tumors,^26^ we observed that the papillary growth pattern was more frequently found in Wt tumors in this study. Most InvAd-MV tumors were Wt (92.9%), which was consistent with a previous report that InvAd-MV tumors were strongly associated with KRAS mutations.^15^

Yanagawa et al examined *EGFR* gene mutations in 241 resected lung adenocarcinoma specimens (49.6%) from a group of 486 patients. They compared histological subtypes of adenocarcinoma between 131 Mt tumors (including 80 cases of Ex21 [33.2%] and 48 cases of Ex19 [19.9%]) and 110 Wt tumors (45.6%). They reported that 62% of AIS, 60% of MIA, 77% of lepidic, 49% of acinar, 50% of papillary, 28% of solid, and 43% of micropapillary subtype tumors were Mt lesions,^16^ which was similar to our results. However, the frequency of InvAd-LP tumors was lower (it was approximately 20%), and the frequency of InvAd-AP and InvAd-SP tumors was higher (it was approximately 10%), in our study (Figure 1) than in previous reports. These results reflect differences in the distribution of EGFR status; our study included a greater proportion of Wt and Ex19 tumors (46.9% and 20.9%, respectively) and a smaller proportion of Ex21 tumors (29.4%) than the study by Yanagawa et al.

Zhang et al^25^ reported that Mt tumor status was associated with AP lung adenocarcinoma, while other studies reported that Mt tumors were associated with MP lung adenocarcinoma.^23,26–29^ Russel et al^39^ reported that 22% of stage III (N2) lung cancer were Mt tumors, and acinar and MP lung adenocarcinoma was associated with Mt. Differences in the histologic subtypes of adenocarcinoma associated with Mt tumors were possibly observed because of differences in the ratios of Ex19 and Ex21 mutations; these differences stemmed from sample size, racial differences, and lung cancer stage. Few studies have compared adenocarcinoma histologic subtypes according to Ex21 and Ex19 status in a large sample size. Yoshizawa et al^15^ reported that there were no significant differences in histologic subtypes of adenocarcinoma when they compared 48 Ex19 tumors and 36 Ex21 tumors. Villa et al^24^ compared 22 Ex19 tumors and 12 Ex21 tumors and found that Ex21 was associated with LP adenocarcinoma; however, other differences in histologic pattern were not observed.

Vascular invasion occurred most frequently in Wt tumors (22.7%; *P* < 0.05) and least frequently in Ex21 tumors. This was consistent with the conclusion that Wt tumors were high-grade malignancies and Ex21 tumors were low-grade malignancies. However, lymph node metastasis was identified most frequently in Ex19 tumors (Table 2). Further studies are necessary to examine whether tumors with Ex19, which is indicative of malignancy that is an intermediate grade (between Wt and Ex21), present with lymph node metastasis at the highest frequency of the 3 EGFR statuses. Moreover, further biomolecular examination is necessary to evaluate correlations between the 3 EGFR statuses and cancer cell invasiveness.

Previous reports demonstrated that Ex21 and Ex19 exhibited different behavior in advanced lung cancer.^1,10,12,13,40^ Rosell et al^13^ reported that Ex19 tumors had a better response rate to TKIs than Ex21 tumors (odds ratio, 3.08; *P* = 0.001). Furthermore, Riely et al^12^ reported that patients with Ex19 tumors had a longer overall survival than patients with Ex21 tumors after erlotinib or gefitinib treatment (34 vs 8 months; *P* = 0.01). Fang et al^14^ reported that progression-free survival was longer in patients with Ex19 versus Ex21 tumors treated with first-line platinum-based chemotherapy (*P* = 0.007). It was questionable whether Ex21 tumors, which are characterized as low-grade adenocarcinoma that is anchored by the lepidic growth pattern, progressed to advanced lung cancer that was resistant to TKIs and chemotherapy. Further evaluation is necessary to examine the factors that conclusively differentiate unresectable advanced lung cancer from resectable lung cancer in patients harboring the same EGFR mutation.

Ex19 mutations varied in type, and they included in-frame deletions and mixed insertion/substitutions; delE746_760 and delL747_P753insS were the 2 most common Ex19 mutations. Response to TKIs varies according to the subtype of Ex19.^11^ The fragment analysis used in this study to examine Ex19 was not able to detect the number of deleted nucleotides and the start codon in order to identify the subtype of Ex19 mutation. A future study is needed to evaluate correlations between Ex19 mutation subtype and clinicopathological features.

Among the clinical stage I lung adenocarcinoma specimens, Ex21 tumors were predominately low-grade adenocarcinomas with a lepidic growth pattern. Moreover, Wt tumors were frequently high-grade adenocarcinomas that mainly comprise solid and papillary components with vascular invasion. Ex19 tumors appeared to be intermediate-grade adenocarcinomas with characteristics that were intermediate to Ex21 and Wt tumors. The pathological characteristic of the pulmonary lesion and the malignancy grade may be predicted preoperatively if the EGFR status of the lesion can be confirmed using small samples. The correlation between EGFR mutation status and clinicopathological features identified in this study suggest that the prognosis may differ according to the EGFR status among clinical stage I lung adenocarcinomas, and that further study is necessary.
